# Facile and versatile ligand analysis method of colloidal quantum dot

**DOI:** 10.1038/s41598-021-99358-x

**Published:** 2021-10-06

**Authors:** Jin Hae Kim, Hyokeun Park, Tae-Gon Kim, Hyunmi Lee, Shinae Jun, Eunha Lee, Woo Sung Jeon, Jaegwan Chung, In-Sun Jung

**Affiliations:** 1 Daegu Gyeongbuk Institute of Science and Technology, 333 Technojungang-daero, Hyeonpung-eup, Dalseong-gun, Daegu, 42988 Republic of Korea; 2 Samsung Advanced Institute of Technology, Samsung Electronics Co., Ltd, 130 Samsung-ro, Yeongtong-gu, Suwon-si, Gyeonggi-do 16678 Republic of Korea

**Keywords:** Materials chemistry, Analytical chemistry, NMR spectroscopy

## Abstract

Colloidal quantum-dots (QDs) are highly attractive materials for various optoelectronic applications owing to their easy maneuverability, high functionality, wide applicability, and low cost of mass-production. QDs usually consist of two components: the inorganic nano-crystalline particle and organic ligands that passivate the surface of the inorganic particle. The organic component is also critical for tuning electronic properties of QDs as well as solubilizing QDs in various solvents. However, despite extensive effort to understand the chemistry of ligands, it has been challenging to develop an efficient and reliable method for identifying and quantifying ligands on the QD surface. Herein, we developed a novel method of analyzing ligands in a mild yet accurate fashion. We found that oxidizing agents, as a heterogeneous catalyst in a different phase from QDs, can efficiently disrupt the interaction between the inorganic particle and organic ligands, and the subsequent simple phase fractionation step can isolate the ligand-containing phase from the oxidizer-containing phase and the insoluble precipitates. Our novel analysis procedure ensures to minimize the exposure of ligand molecules to oxidizing agents as well as to prepare homogeneous samples that can be readily analyzed by diverse analytical techniques, such as nuclear magnetic resonance spectroscopy and gas-chromatography mass-spectrometry.

## Introduction

**Figure 1:**
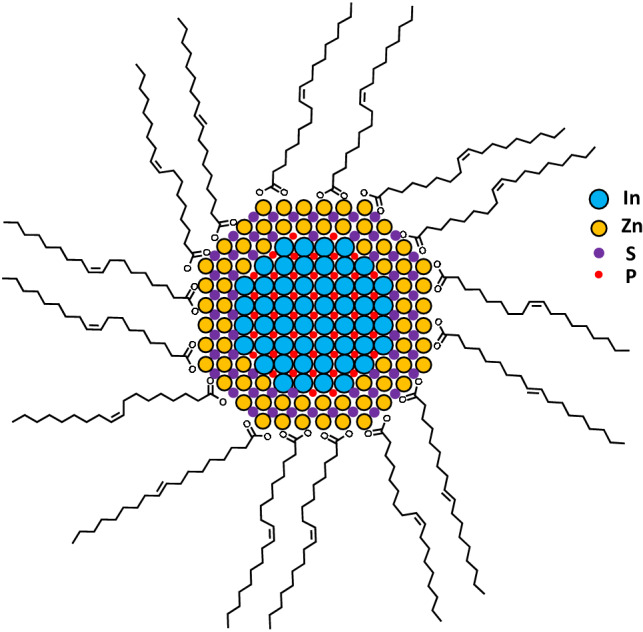
Schematic structure of an oleic-acid (OA) capped InP/ZnS QD with an inorganic InP/ZnS crystalline core (collection of spheres colored as designated) and OA molecules as passivating ligands (black sticks).

Colloidal quantum-dots (QDs) are nano-sized crystalline semiconductors. By modulating their size, composition, and surface-passivating molecules, various semiconducting features can be imparted to these materials, thus rendering them versatile for many applications such as light-emitting devices, display devices, solar cells, and sensors^1–5^. QDs are synthesized by modulating the inorganic precursors and the associated organic molecules, reaction temperature, and time; the resulting QD is composed of an inorganic nano-crystalline core surrounded by the organic ligand molecules (Fig. 1)^6^. The ligands of QDs play important roles of shaping the structure of the core, passivating and protecting the cores after their growth, and providing additional physical and chemical functionalities to QDs^7^. Many studies have been conducted on developing novel procedures of modulating the ligand composition of QDs to impart or maximize the desired physicochemical properties and functionalities^7,8^.

Ligand optimization is one of the most promising strategies to improve the performance and stability of QDs^7,8^. However, analytical tools available to detect and characterize various ligand molecules are still limited. The procedures of QD syntheses involve harsh conditions (e.g., high temperature, pressure, and highly reactive species) that often lead to the accumulation of by-products on the surface of QD particles^9,10^. In addition, although ligand-exchange processes are frequently used to impart novel chemical functionalities to QDs, it is still difficult to examine the final status of ligands due to the lack of appropriate analytical methodologies.

Various techniques and protocols^11,12^ have been developed for ligand analysis to date; among them, nuclear magnetic resonance (NMR) spectroscopy^13,14^ and gas-chromatography mass-spectrometry (GC–MS)^15^ have been notably powerful to elucidate the identity and composition of organic ligands on the QD surface. However, information from either technique is often limited owing to its intrinsic technical hurdles. Signal quality of solution-state NMR spectroscopy depends on isotropic tumbling rates of target molecules. Although ligand molecules are as such small and tumble fast enough to yield sharp signals, the tumbling of ligand molecules bound on QD is much slower, making the corresponding NMR signals broad and unsuitable even for simple 2-dimensional (2D) NMR experiments^13^. Similarly, because GC–MS detects organic molecules in a gas phase, ligand molecules first need to be easily transformed into the gaseous state for GC–MS analysis to be feasible. QD ligands are, however, often tightly bound to the nanoparticle surface, which prevents them from being gasified. This challenge is sometimes overcome by inducing ligand dissociation through chemical modification (e.g., ligand derivatization) prior to GC analysis; however, this procedure compromises the original chemical integrity of the ligands^16^.

In order to overcome this challenge, we devised a novel protocol of using oxidizing agents to detach organic molecules from the inorganic core or shell surface. Although several procedures in which ligands on QD are detached for their analysis were reported^17–19^, their applications were often limited to certain types of nanoparticles or ligand molecules. Based on previous studies that have demonstrated that oxidation disrupts the interaction between the ligands and nanocrystal^20^, we developed a protocol of oxidizing the nanocrystals and subsequently separating the ligand-containing fraction. Among various oxidizing agents, we found that hydrogen peroxide (H_2_O_2_) is an efficient heterogeneous catalyst to detach most ligands from inorganic parts, while being mild enough to minimize the disruption of their chemical integrity. Owing to phase difference, H_2_O_2_ could be easily removed from the ligand-containing organic phase by a simple fractionation step, further ensuring minimal exposure of ligands to oxidation. Previous studies have shown that H_2_O_2_ is capable of etching nanoparticles to enhance their functionalities without compromise in performance^21,22^. We applied our method to analyze the ligands of InP/ZnS QD samples. Despite their overall inferior performance and stability, InP-based QDs have attracted significant interest due to their suitability for environment-friendly application^23,24^.

## Results and discussion

**Figure 2:**
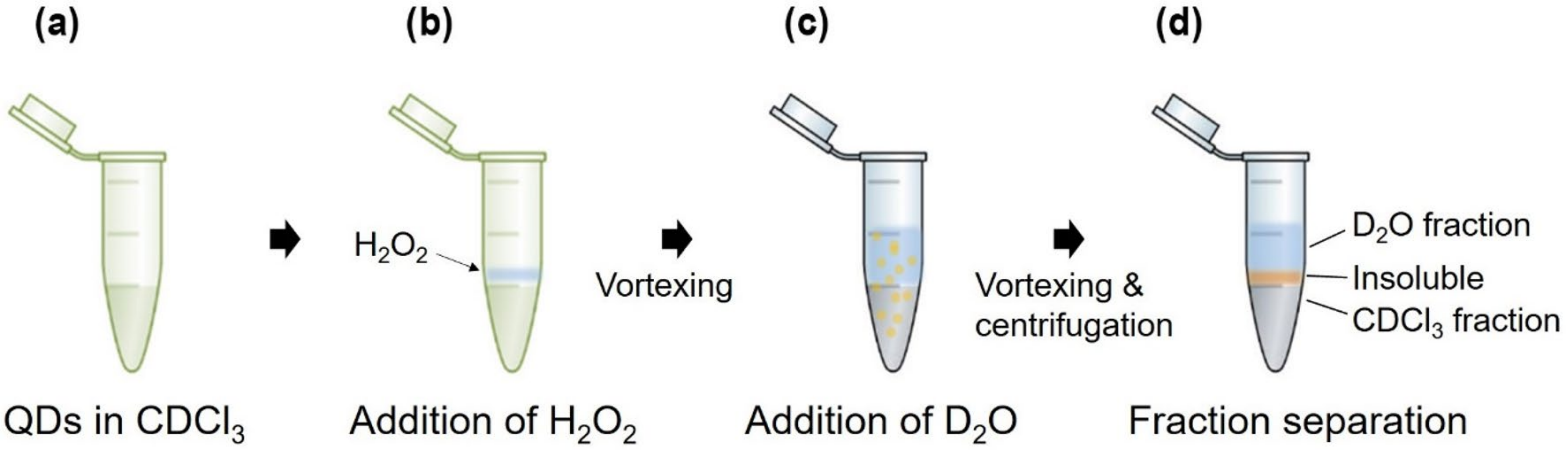
The ligand analysis procedure involving the use of H_2_O_2_ as a heterogeneous oxidizing agent. First, the QDs dispersed in CDCl_3_ (**a**) are treated with H_2_O_2_ (**b**) and vortexed vigorously. Green color originating from the QDs is retained until step (**b**). Upon reaction under ambient condition, the sample loses its green color, leading to an insoluble layer (precipitate). In order to separate the fraction containing organic ligands from that containing H_2_O_2_ and H_2_O, D_2_O is added to the sample, and the sample is again vortexed vigorously (**c**). Subsequently, the sample is separated into three fractions via centrifugation: the D_2_O fraction, insoluble fraction, and CDCl_3_ fraction (**d**). The final soluble fractions are transparent and colorless.

**Figure 3:**
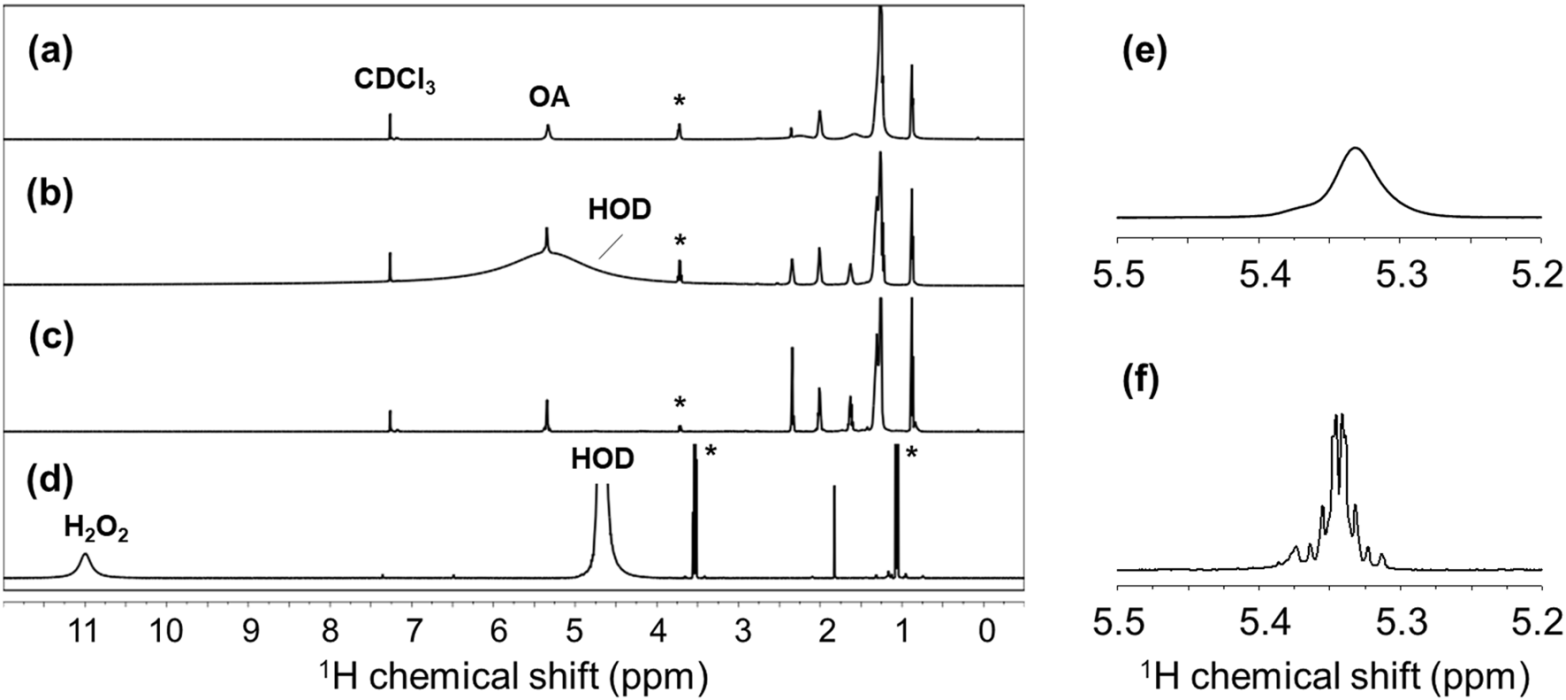
^1^H NMR spectra of InP/ZnS-OA: (**a**) as-prepared sample in CDCl_3_, (**b**) after treatment with 50 μL of H_2_O_2_, (**c**) CDCl_3_ fraction, and (**d**) D_2_O fraction. The CDCl_3_ and D_2_O fractions were obtained after the centrifugation of H_2_O_2_-treated samples. The ^1^H signals at 5.35 ppm (vinyl protons of OA) for the as-prepared sample in CDCl_3_ (**e**) and for the CDCl_3_ fraction sample separated after H_2_O_2_ treatment (**f**) are shown to stress that H_2_O_2_ treatment and phase fractionation effectively reduced the signal width of ligand molecules. Asterisks (*) indicate the signals of residual ethanol from purification procedure of QD.

The ligand-analysis procedure (Fig. 2) was initiated with the addition of H_2_O_2_ to an oleic-acid capped InP/ZnS QD (InP/ZnS-OA) sample dispersed in organic solvents, such as CDCl_3_. Upon oxidation, discoloration of the sample and subsequent precipitation of an insoluble material were observed (Fig. 2c). After bleaching, D_2_O was added to the sample, and the sample was centrifuged to obtain three different fractions: a CDCl_3_ fraction, a D_2_O fraction, and an insoluble layer (Fig. 2d and Fig. S1). By X-ray photoelectron spectroscopy (XPS), we confirmed that the insoluble layer mostly contained oxidized inorganic material from the core and shell (such as ZnSO_4_ and InPO_4_; Fig. S2). We also found that the D_2_O addition and fraction separation steps are critical for high-resolution ligand analysis. D_2_O plays an important role to retrieve excess H_2_O_2_ from the CDCl_3_ fraction and contain it to the D_2_O fraction in a diluted state, thus minimizing the exposure of ligands to H_2_O_2_ as organic molecules were partitioned into the CDCl_3_ fraction. Moreover, D_2_O addition increased the effective volume of H_2_O_2_-containing fraction, making its removal easy with a simple pipetting action. Fraction separation procedure is important to remove most H_2_O_2_ and H_2_O molecules from the CDCl_3_ fraction, which prevents signal overlapping and broadening for high-resolution 1D/2D NMR analyses. It is also advantageous that water-soluble materials, if any, were separated from CDCl_3_-soluble ligands, simplifying the subsequent ligand characterization (Fig. 3).

The final CDCl_3_ fraction provided sharp ^1^H NMR signals (Fig. 3e vs f.) that were further analyzed by multi-dimensional NMR techniques (see below). Notably, we were still able to retrieve active and stable QDs by conducting fraction separation shortly after H_2_O_2_ addition, indicating that H_2_O_2_ had indeed been removed from the QD-containing CDCl_3_-fraction; the same H_2_O_2_-treated QD sample was found to completely bleach out when incubated without fraction separation. In addition, we observed that the ^1^H NMR signal originating from H_2_O was significantly weak for the CDCl_3_ fraction, while the NMR signal of H_2_O was clearly observed with the H_2_O_2_-treated QD samples not subjected to fraction separation (Fig. 3b vs c). Consistently, only the D_2_O fraction exhibited the ^1^H NMR signal for H_2_O_2_ (Fig. 3d)^25^. We also confirmed that the intensities of ^1^H OA signals from the CDCl_3_-extracted fraction correlated well with the added amount of QDs (Fig. S3). With the calibration curves of OA (Fig. S3b), it is possible to quantify ligand molecules and determine the surface coverage or ligand composition at a higher accuracy (the procedure for surface coverage analysis and the detailed calculation results on the QD sample are described in supplementary method).

**Figure 4:**
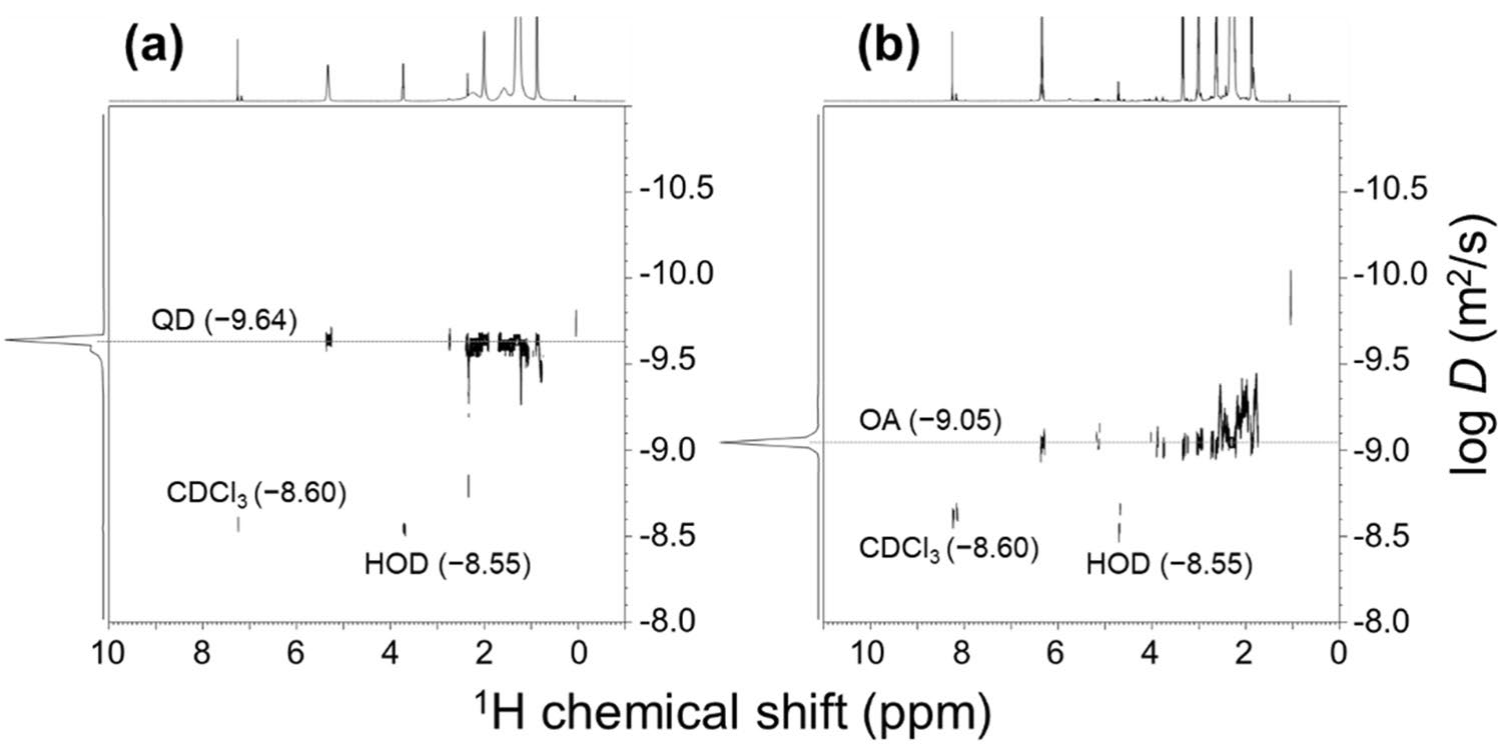
2D-DOSY NMR spectra of InP/ZnS-OA: (**a**) before and (**b**) after treatment with 50 μL of H_2_O_2_ and CDCl_3_ fraction separation. The vertical axis shows the diffusion coefficient (log *D*). The measured diffusion coefficients for OA and solvent signals are denoted in the figure.

**Figure 5:**
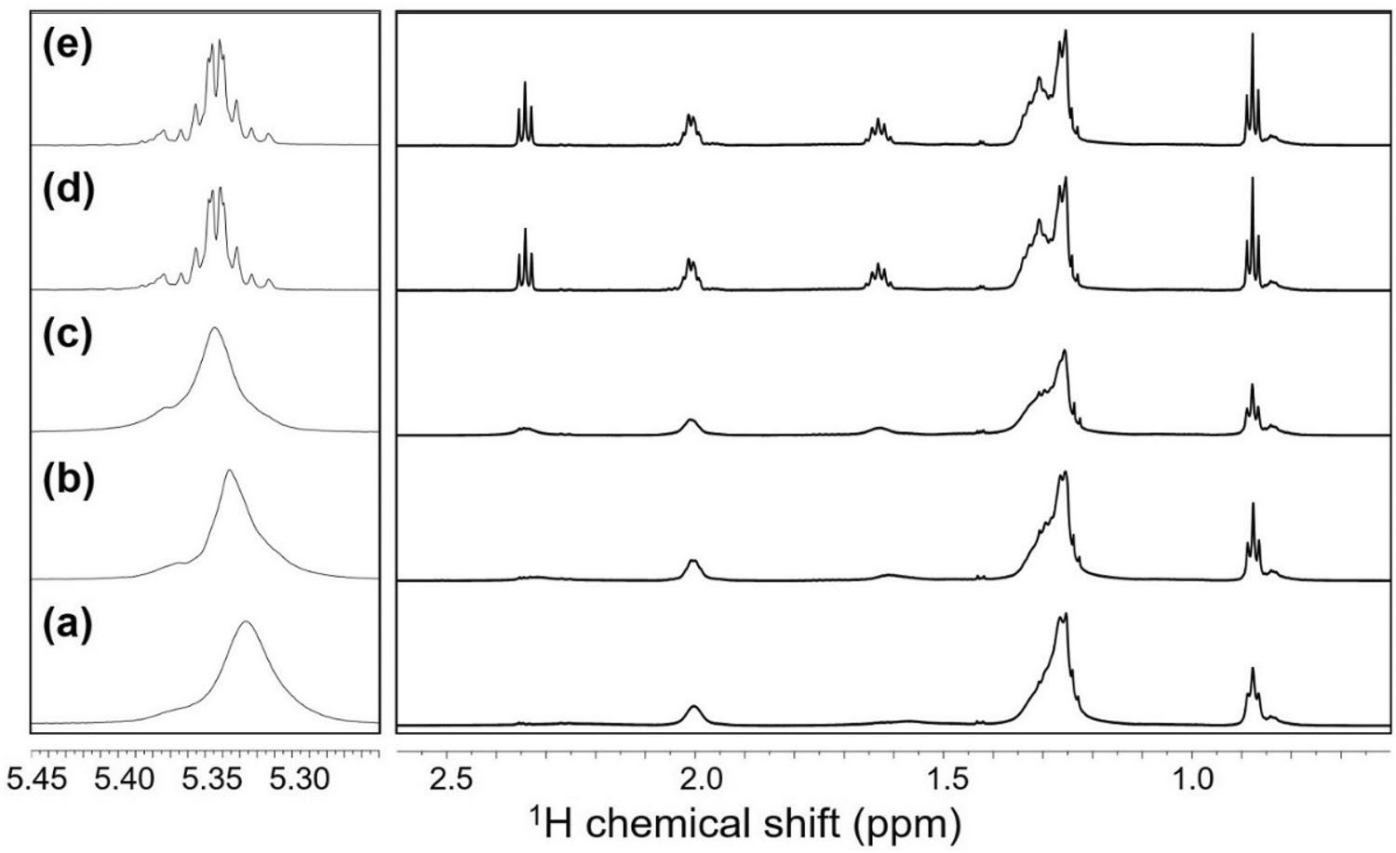
^1^H NMR spectra of InP/ZnS-OA with adding different amounts of H_2_O_2_, (**a**) no H_2_O_2_, (**b**) 5 μL, (**c**) 15 μL, (**d**) 50 μL, and (**e**) 100 μL, and then extracted into the CDCl_3_ fraction.

Next, we examined whether the ligand signals of the CDCl_3_ fraction were indeed from free ligands (not from QD-bound ligands). 2D DOSY is a useful tool to measure the hydrodynamic radius of molecules in the solution and distinguish the molecular weight or size of molecules in different mixtures^26,27^. By employing this technique, we confirmed that the bound OA ligands (log *D*, − 9.64) on the QD surface in the as-prepared sample diffuse slower than the free ligands (log *D*, − 9.05) in the CDCl_3_ fraction of the H_2_O_2_-treated QD sample (Fig. 4). This result indicates that H_2_O_2_ treatment liberated the ligands from the high molecular-weight inorganic particle, which is also consistent with significant NMR signal sharpening and discoloration of the H_2_O_2_-treated sample. Subsequently, we compared the spectral quality of the series of CDCl_3_-fraction samples obtained after treatment with different amounts of H_2_O_2_. We added 0, 5, 15, 50, and 100 μL of H_2_O_2_ to a series of QD samples (same amount), and collected the ^1^H NMR spectra of their CDCl_3_-fraction samples (Fig. 5). This spectral comparison revealed that the relative portion of free ligands increased with the amount of H_2_O_2_ up to 50 μL H_2_O_2_, while there was no significant difference in the spectra with further increase in its quantity (Fig. 5d vs. e), implying minimal artifact from excess treatment of H_2_O_2_, if any. In addition, we tested whether the incubation reaction time with H_2_O_2_ (i.e., the time interval between H_2_O_2_ addition and fraction separation) affects the ligand dissociation and NMR spectral quality. As expected, ligand signals sharpened with the reaction time (Fig. S4), which is again consistent with time-dependent QD oxidation and subsequent ligand dissociation. Notably, we also tested whether addition of D_2_O may affect H_2_O_2_-mediated oxidation of QD (Fig. S5). From this trial, we confirmed that H_2_O_2_ cannot efficiently oxidize QD in a D_2_O-diluted condition, validating that the addition of D_2_O contributes to regulation of H_2_O_2_-mediated oxidation as well as prevention for over-oxidation of ligand molecules.

This novel protocol of ligand sample preparation is also good for GC–MS analysis. The routine GC–MS ligand analysis procedure often involves the compromise of the chemical integrity of ligand molecules. In contrast, the sample prepared with the present protocol only contains free organic ligands with minimal modification, if any, enabling straightforward and reliable GC–MS analysis (Fig. S6). Notably, GC–MS could be used to detect palmitic acid (PA), stearic acid (SA), and trioctylamine (TOA) in addition to OA. Due to the chemical similarity between these molecules, 1D ^1^H NMR could not be used to resolve PA, SA, or TOA signals from the OA signals. By employing this GC–MS-based approach, we found that two distinctive compositions of the ligands (particularly, PA and OA) in two different batches (QD 1 and QD 2) of InP/ZnS-OA could be identified (Fig. S6). In addition, we were able to determine relative response factors^28^ for OA, PA, and TOA (Fig. S7), which could be subsequently applied to analyze the GC–MS chromatograms to have accurate quantification analysis of QD ligands (Fig. S6e).

**Figure 6:**
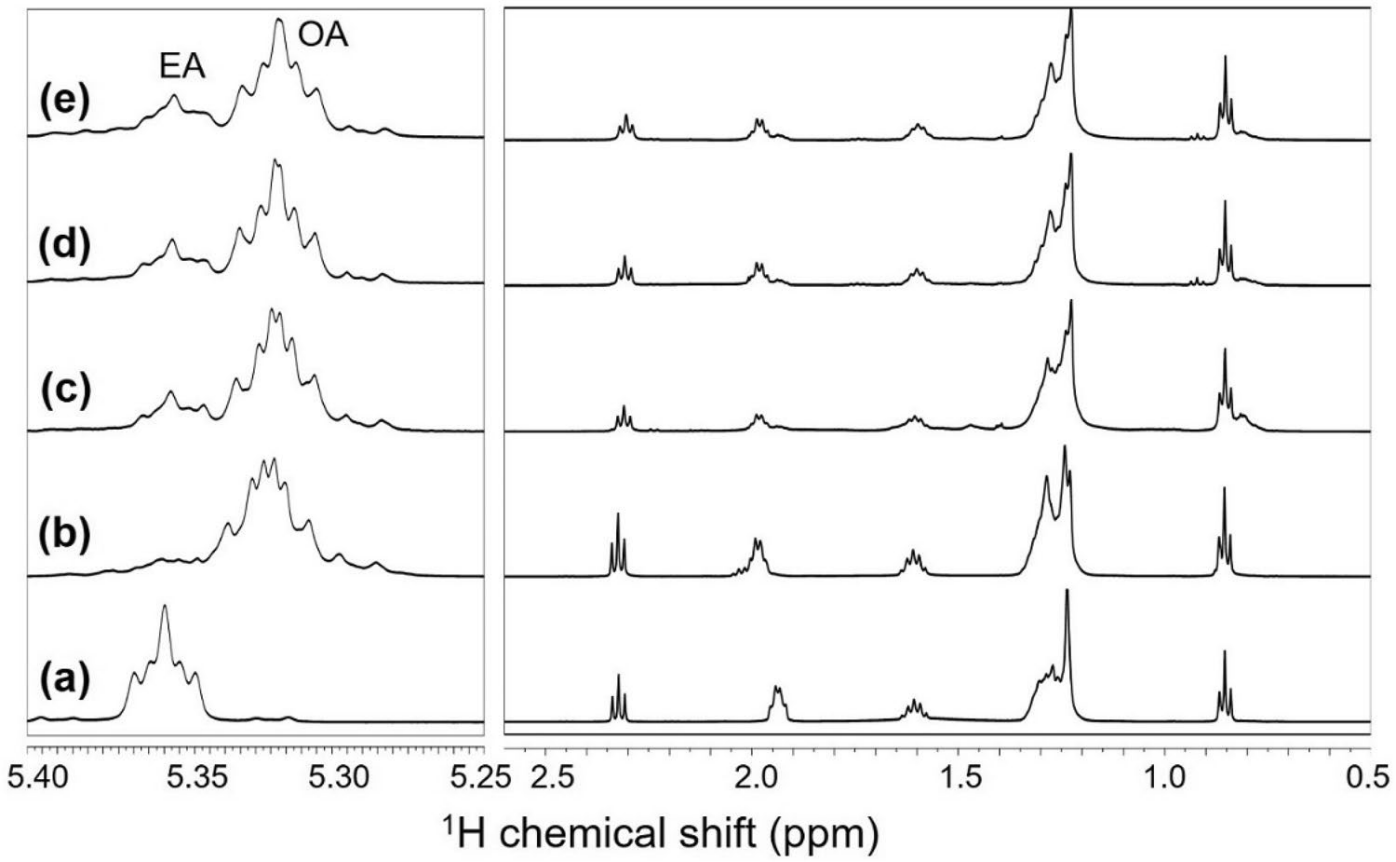
^1^H NMR spectra of the CDCl_3_ fractions separated from the H_2_O_2_-treated samples of (**a**) elaidic acid (EA), (**b**) oleic acid (OA), (**c**) CdS-OA QD, (**d**) InP/ZnS-OA QD batch #1, and (**e**) InP/ZnS-OA QD batch #2. The extra peaks (marked as ‘EA’) near the signals of OA correspond to the *trans*-isomer of OA, i.e., EA [refer Fig. S8 for ^1^H NMR spectra of the as-prepared (without H_2_O_2_ treatment) samples].

Finally, we decided to evaluate whether the new approach can be applied to characterize ligands de novo. Due to the lack of an appropriate analytical methodology, identification and characterization of ligands without prior information (e.g., precursors used for QD synthesis) has been challenging. As we were able to consistently observe a set of distinctive ^1^H signals near the signals of OA with our QD samples (e.g., the overlapped signal on the shoulder of the OA signal at ~ 5.3 ppm; Fig. S8), we decided to identify the ligand molecule presenting these signals. While the signals from ligands, in their intact QD-bound state, are broad and not amenable for further analysis, the novel protocol enabled us to obtain sharper and higher-resolution signals from the CDCl_3_ fraction of the H_2_O_2_-treated QD samples (Fig. 6). We collected a series of 2D NMR spectra, such as ^1^H–^1^H TOCSY and ^1^H–^13^C HSQC (Fig. S9), with the CDCl_3_ fraction, and found that this spectral feature arises from the *trans*-isomer of OA, i.e., elaidic acid (EA; Fig. 6).

We confirmed the presence of EA by comparing the data with the NMR data of a sample containing only EA (Fig. 6a) and by obtaining the ion chromatograms and mass spectra of extracted EA and OA using a highly polar column (Fig. S10)^29^. In order to determine how EA was formed, we tested whether high temperature, as in the QD synthesis procedures, facilitated OA isomerization. Indeed, we observed that the nucleation/growth of QDs at 120 °C induced time-dependent formation of EA, suggesting that EA on QDs might have been originated from OA (data not shown)^30^.

## Conclusion

We developed a novel methodology of H_2_O_2_-induced oxidation by which we induced the efficient liberation of ligands, which upon subsequent phase fractionation becomes a sample suitable for various analytical techniques. We confirmed that H_2_O_2_ as a heterogeneous catalyst did not compromise the chemical integrity of ligand molecules, thus demonstrating the accuracy and versatile applicability of the present method. We also found that the D_2_O addition and fraction separation step was effective to remove H_2_O_2_ from the ligand-containing apolar fraction, thus preventing any possible deformation of organic ligands, as well as to fractionate polar and apolar ligand molecules into separate fractions, which is advantageous to reduce the complexity in the ligand mixtures. Overall, our ligand sample preparation procedure effectively enhances the overall resolution and sensitivity at the subsequent ligand analysis. We would like to stress that this strategy is wildly compatible with various ligand molecules having different chemical properties. In our follow-up ligand analysis studies that will be reported elsewhere, we also found that amine and sulfhydryl ligands can be analyzed by the present method; these ligands were detected as a form of amide and disulfide, sulfoxide, or sulfonate, respectively.

In this work, we particularly focused on applying NMR spectroscopy and GC–MS for sample analysis to prove that, upon being combined with the novel sample preparation procedures, these two techniques are capable of exerting their full analytical strengths for the identification and characterization of diverse organic molecules. Notably, we were able to determine the structural isomerization of OA molecules possibly during the QD synthesis, exemplifying the good sensitivity and resolvability of the developed methodology for organic ligands. Notably, the samples prepared with the presented procedure can be analyzed not only with NMR and GC–MS but also with other analytical techniques such as LC–MS, ICP, and IR. Compared to previous attempts to analyze ligands in the QD-bound state, our new analytical procedure may greatly advance our understanding of the ligand composition of QD samples, ligand degradation during the QD synthesis and subsequent manipulation, and the correlation between the nature of ligands and the QD performance.

## Methods

*Synthesis of InP/ZnS nanocrystals.* Indium acetate and oleic acid were dissolved in 1-octadecene in a 200 mL reaction flask under vacuum at 120 °C for 1 h. The molar ratio of indium to oleic acid (OA) was set to 1:3. Then, N_2_ gas was flown into the flask and the reaction mixture was heated to 280 °C. Subsequently, a mixed solution of tris(trimethylsilyl) phosphine (TMS_3_P) and trioctylphosphine was injected quickly, and the reaction was allowed to proceed for 20 min. The reaction mixture was then cooled rapidly to room temperature and acetone was added to precipitate the nanocrystals, which were then separated by centrifugation and dispersed in toluene to obtain a suspension of the InP core nanocrystals. The amount of TMS_3_P used was ~ 0.75 mol per mole of indium. For coating a ZnS shell, in a 200 mL reaction flask, zinc acetate and OA were dissolved in trioctylamine and the solution was heated under vacuum at 120 °C for 10 min. Then, N_2_ was flown into the reaction flask. While the resulting solution was heated to 280 °C, a toluene dispersion of the InP cores was injected into it followed by the injection of 1-dodecanethiol. The reaction was carried out for 60 min. The total amount of S used per mole of indium was set to 6 mol. An excess amount of ethanol was added to the final reaction mixture containing the resulting InP/ZnS nanocrystals, which was then centrifuged, the supernatant was discarded, and the precipitate was dried to obtain the QD sample in a powder form. The UV–Vis absorption and photoluminescence spectra and the transmission electron microscopy image of the as-prepared InP/ZnS-OA QD samples are shown in Fig. S11.

*Oxidative treatment of QDs for ligand analysis.* Ligand analysis procedure was initiated with the dispersion of 10 mg of an oleic-acid capped InP/ZnS QD (InP/ZnS-OA) sample in 500 μL of CDCl_3_. This sample was treated with a desired amount of H_2_O_2_, vigorously vortexed, and incubated at the ambient condition. During reaction with H_2_O_2_ incubation, the sample was repeatedly vortexed in order to mix the reaction mixture thoroughly. Once the QD sample was incubated for a desired period, 500 μL of D_2_O was added to the sample. The sample was again vortexed vigorously and subsequently centrifuged at 13,000 rpm for 10 min on a table-top centrifuge. After centrifugation, the contents of the tube separated into three fractions: an upper D_2_O layer, a middle insoluble precipitate, and a lower CDCl_3_ layer. Each fraction was separated with a micropipette, and used for subsequent analyses.

*XPS analysis.* The XPS analysis was carried out using Quantum 2000 Scanning ESCA microprobe (manufactured by ULVAC-PHI) with monochromatic Al Kα source (hυ, 1486.60 eV).

*GC–MS analysis.* GC–MS was performed on Thermo Scientific™ ISQ LT GC–MS and Agilent 5977A series MSD. Two columns were used to analyze the ligands: DB5-MSUI (30 m × 250 μm × 0.25 μm) was employed to determine the composition of the ligand mixture, while a highly polar column such as Select FAME (50 m × 250 μm) was employed to separated *cis* and *trans* isomers^29^. Quantitative analyses were carried out by determining the relative response factors of ligand molecules and applying the internal standard method^28^.

*NMR spectroscopy.* The NMR experiments were performed on Bruker NMR spectrometers at 11.7 T and 14.1 T B_0_ field strengths corresponding to ^1^H Larmor frequencies of 500.13 MHz (AVANCE HD III) and 600.1 MHz (AVANCE III), respectively. Bruker 5 mm double resonance broadband probes were used for all the experiments. NMR measurements were performed with 500 μL samples in top-sealed tubes (Nihon Seimitsu Kagaku Co. Ltd) so to minimize the effects of sample evaporation and air-exposure. 2D diffusion-ordered spectroscopy (DOSY) experiments were performed using a stimulated-echo sequence incorporating bipolar gradient pulses and a longitudinal eddy current delay (BPP-LED)^26,27^. The self-diffusion coefficient for residual HDO in D_2_O was found to be 1.9 × 10^–9^ m^2^ s^−1^ at 298 K (log *D* =  − 8.6), which is consistent with the reported data for the reference^27^.

## Supplementary Information

Supplementary Information.
