# Supplementary material for: Facile and versatile ligand analysis method of colloidal quantum dot
Source: Sci Rep. 2021 Oct 6;11:19889. doi: 10.1038/s41598-021-99358-x (PMC8494807; doi:10.1038/s41598-021-99358-x)
Supplement: Supplementary file 1 — Supplementary Information. [file 41598_2021_99358_MOESM1_ESM.docx]

Supplementary Data

**Facile and Versatile Ligand Analysis Method of Colloidal Quantum Dot**

Jin Hae Kim^1,^*, Hyokeun Park^2^, Tae-Gon Kim^2^, Hyunmi Lee^2^, Shinae Jun^2^, Eunha Lee^2^, Woo Sung Jeon^2^, Jaegwan Chung^2^, In-Sun Jung^2,^*

^1^Daegu Gyeongbuk Institute of Science & Technology, 333 Technojungang-daero, Hyeonpung-eup, Dalseong-gun, Daegu 42988, Republic of Korea.

^2^Samsung Advanced Institute of Technology, Samsung Electronics Co., Ltd, 130 Samsung-ro, Yeongtong-gu, Suwon-si, Gyeonggi-do 16678, Republic of Korea.

**
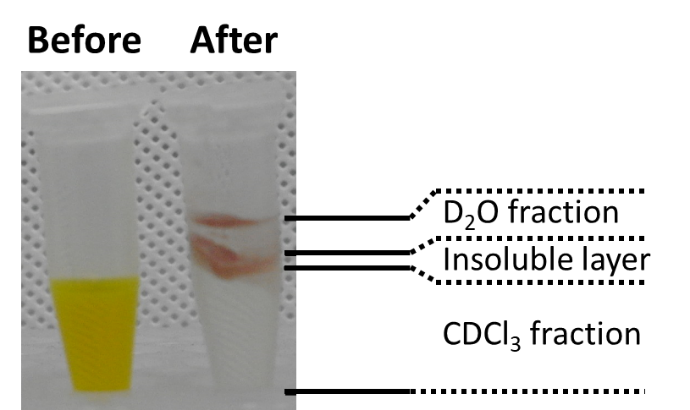
**

**Figure S1.** The photograph of QD samples before and after H_2_O_2_ treatment and phase fractionation. Note the formation of the three separable phases (D_2_O fraction, insoluble layer, and CDCl_3_ fraction) in the ‘After’ sample (right). The volume of the CDCl_3_ fraction was not affected by these procedures, indicating minimal loss (if any) of organic molecules.

**Figure S2.** X-ray photoelectron spectroscopy results of the oxidized insoluble layer of the H_2_O_2_-treated sample (QD 1–4, different batches of the similar QD samples). Note that the oxidized insoluble layer is mostly composed of InPO_4_ and ZnSO_4_.

**
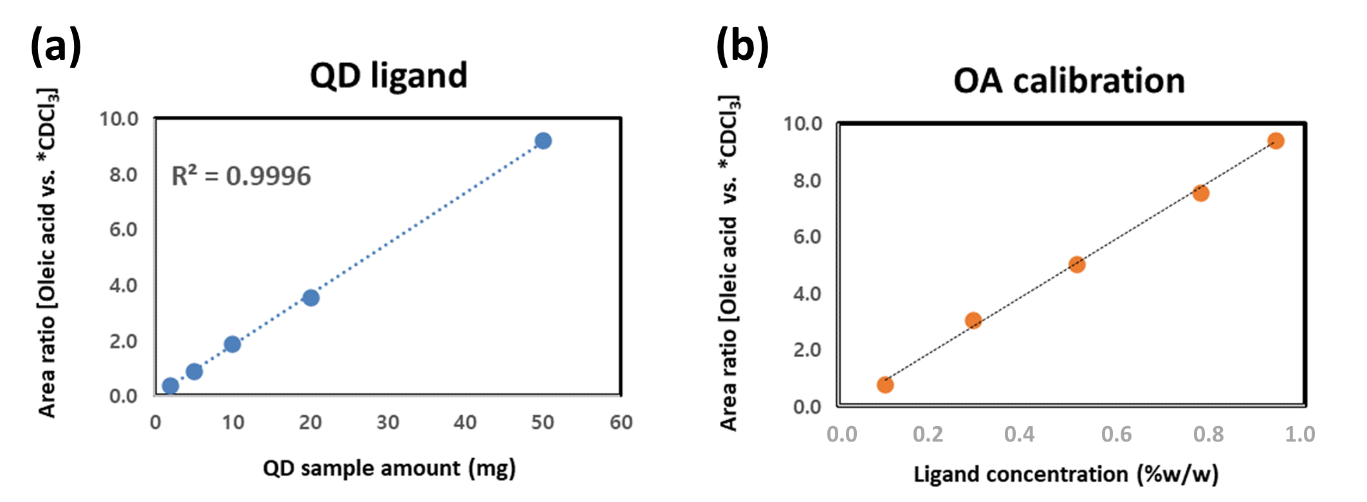
**

**Figure S3.** (a) Correlation between the amount of added QDs and the relative intensity of ^1^H NMR OA signal. InP/ZnS-OA QD samples were prepared with different added amounts (1 mg, 5 mg, 10 mg, 20 mg, and 50 mg), treated with 100 μL H_2_O_2_ (300 μL H_2_O_2_ to 50 mg for an efficient reaction), and then extracted into the CDCl_3_ fraction. The relative intensities of the OA vinyl proton signals at 5.35 ppm were measured against the residual peak of CDCl_3_ (marked as *CDCl_3_). Note that the relative intensity of ^1^H OA signal correlates well with the added amount of QDs, indicating superiority of the current method for ligand quantification. (b) The calibration curve from the standard solutions of OA (%w/w) in CDCl_3_ solution. Along with this, the ligand quantification result as shown in (a) can be translated to the surface coverage and composition of QD ligands in an accurate fashion (refer the supplementary method section for surface coverage analysis procedure and results).

^1^H chemical shift (ppm)

**Figure S4.** ^1^H NMR spectra of the extracted CDCl_3_ fractions from InP/ZnS-OA samples that underwent different reaction durations with 50 μL of H_2_O_2_, (a) without H_2_O_2_ treatment, (b) less than 3 min, (c) 10 min, (d) 15 min, (e) 30 min, and (f) 60 min.


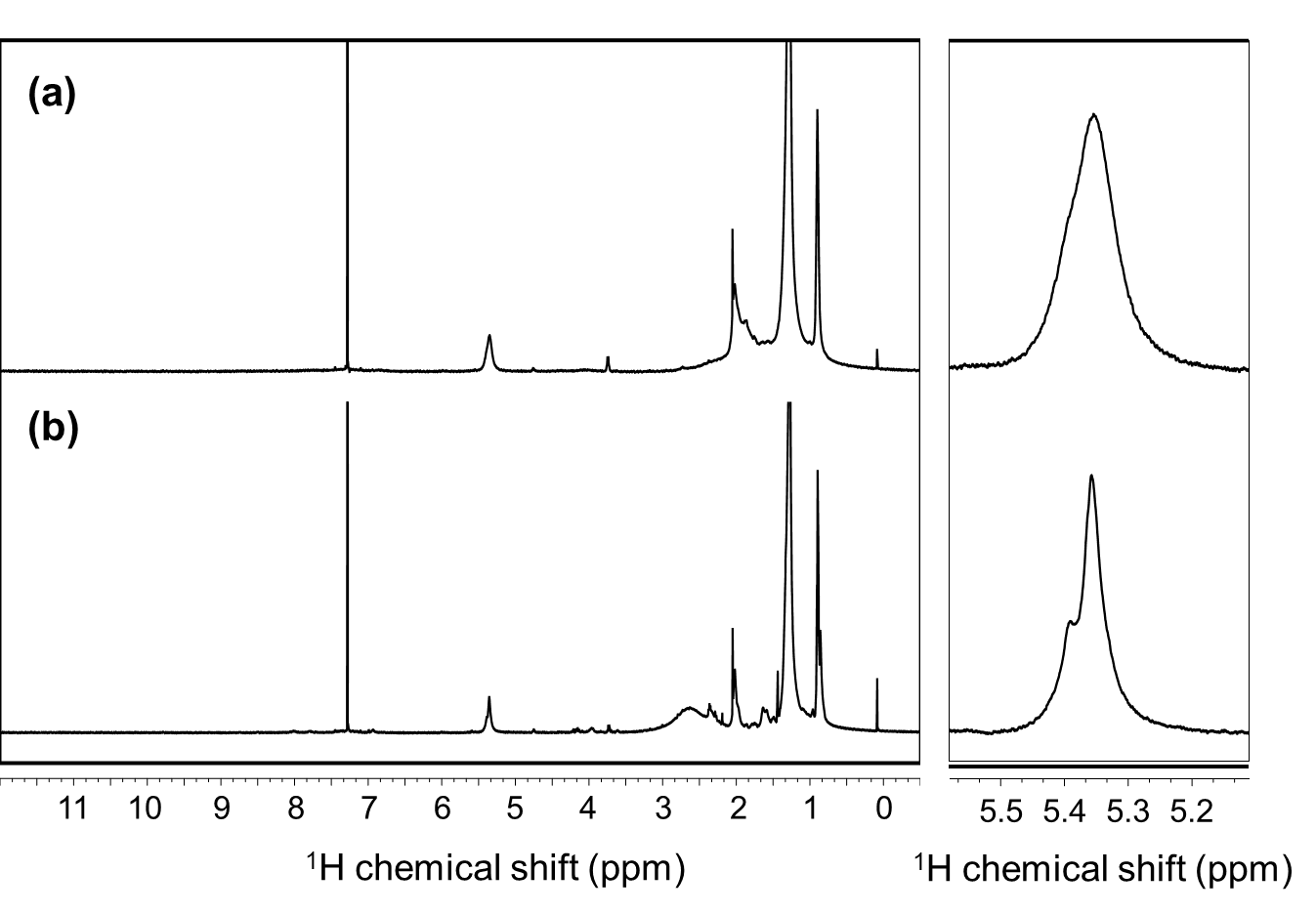


**Figure S5.**  ^1^H NMR spectra of (a) the as-prepared InP/ZnS-OA QD sample in CDCl_3_, and (b) the InP/ZnS-OA QD sample that was treated with the mixture of 50 μL of H_2_O_2_ and 100 μL of D_2_O. It is evident that oxidation-induced ligand detachment process was significantly slowed down in (b).

**
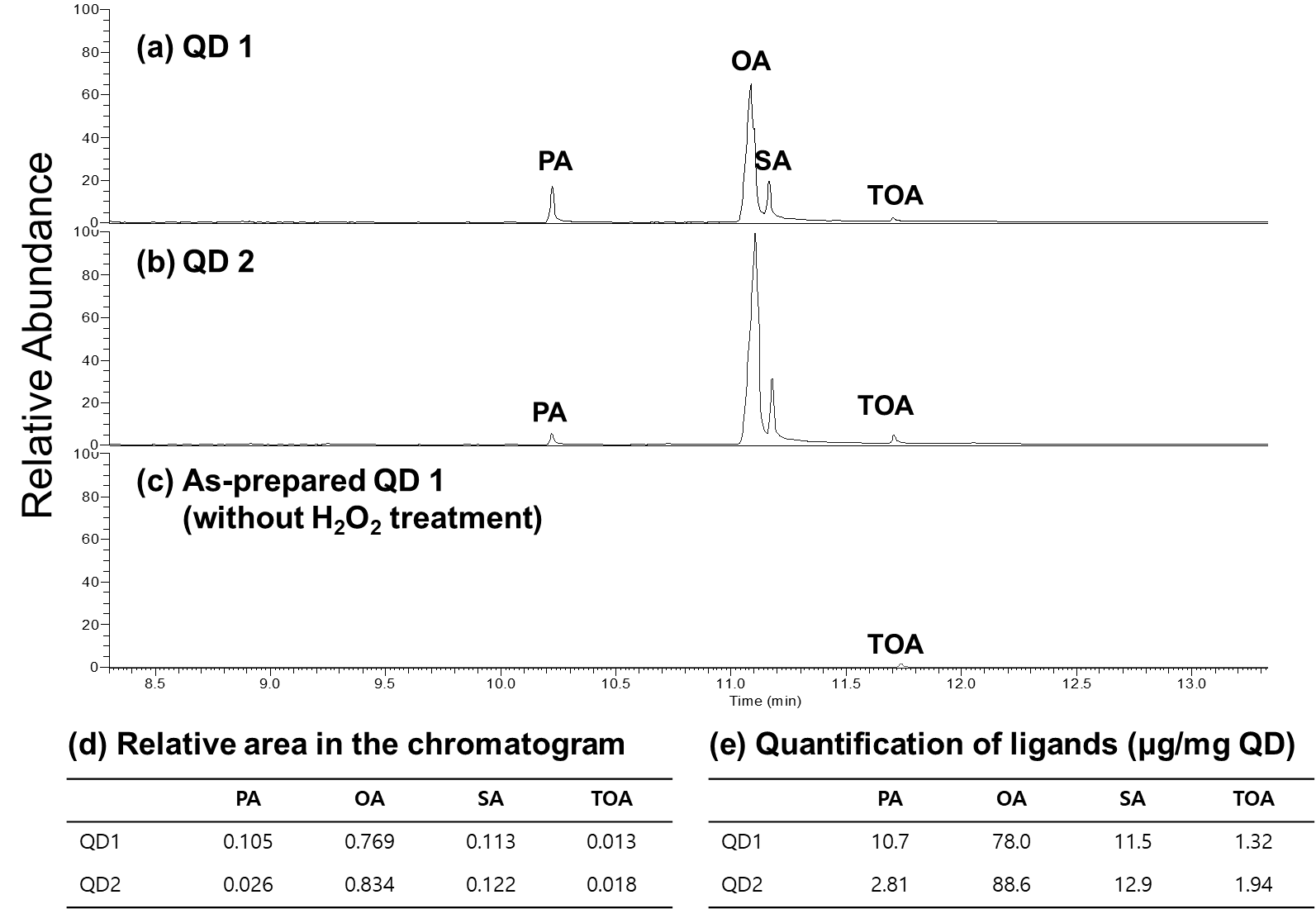
**

**Figure S6.** GC-MS results of the CDCl_3_ fraction from (a) H_2_O_2_-treated InP/ZnS-OA batch #1 (QD 1), (b) H_2_O_2_-treated InP/ZnS-OA batch #2 (QD 2), and (c) as-prepared InP/ZnS-OA batch #1 sample (QD 1) without H_2_O_2_ treatment. The relative area of each ligand peak (d) from GC-MS chromatograms was further analyzed with the relative response factor determination result (refer Figure S7) to have the quantification data for ligands of QD 1 and QD 2 samples (e). The acronyms in the figure stand for palmitic acid (PA), oleic acid (OA), stearic acid (SA), and trioctylamine (TOA). These analyses confirmed that OA is the major ligand of our QD samples. In addition, we verified that, after H_2_O_2_ treatment and phase fractionation, GC-MS could be used to determine the difference in the composition of the ligands in two different batches (QD 1 and QD 2) of InP/ZnS-OA QDs. In contrast, without H_2_O_2_-mediated oxidation, GC-MS failed to detect QD-bound ligand molecules (c).


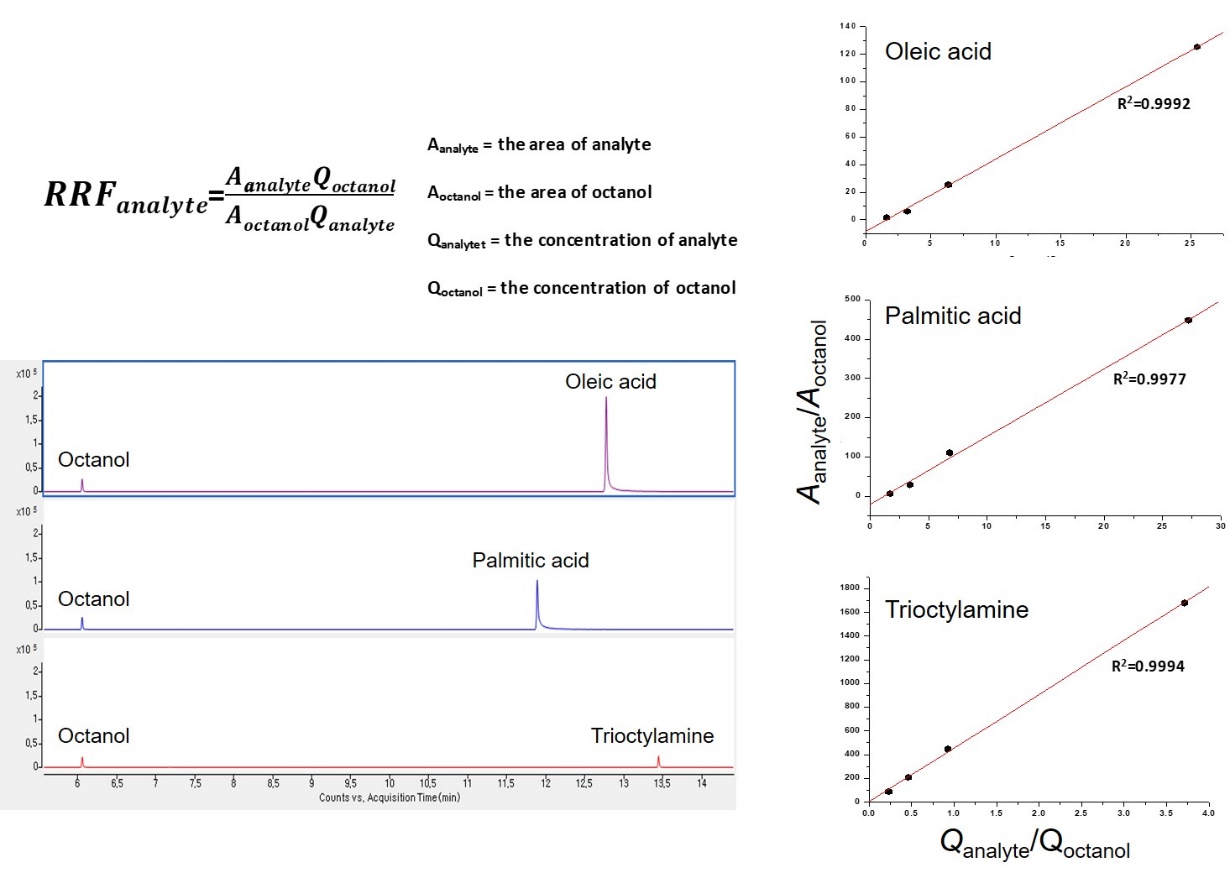


**Figure S7.** Relative response factors (RRFs) for the accurate GC-MS-based quantification of QD ligand molecules. Reliable RRFs for oleic acid, palmitic acid, and trioctylamine were obtained as shown in the right panels (all R^2^ values are higher than 0.99).


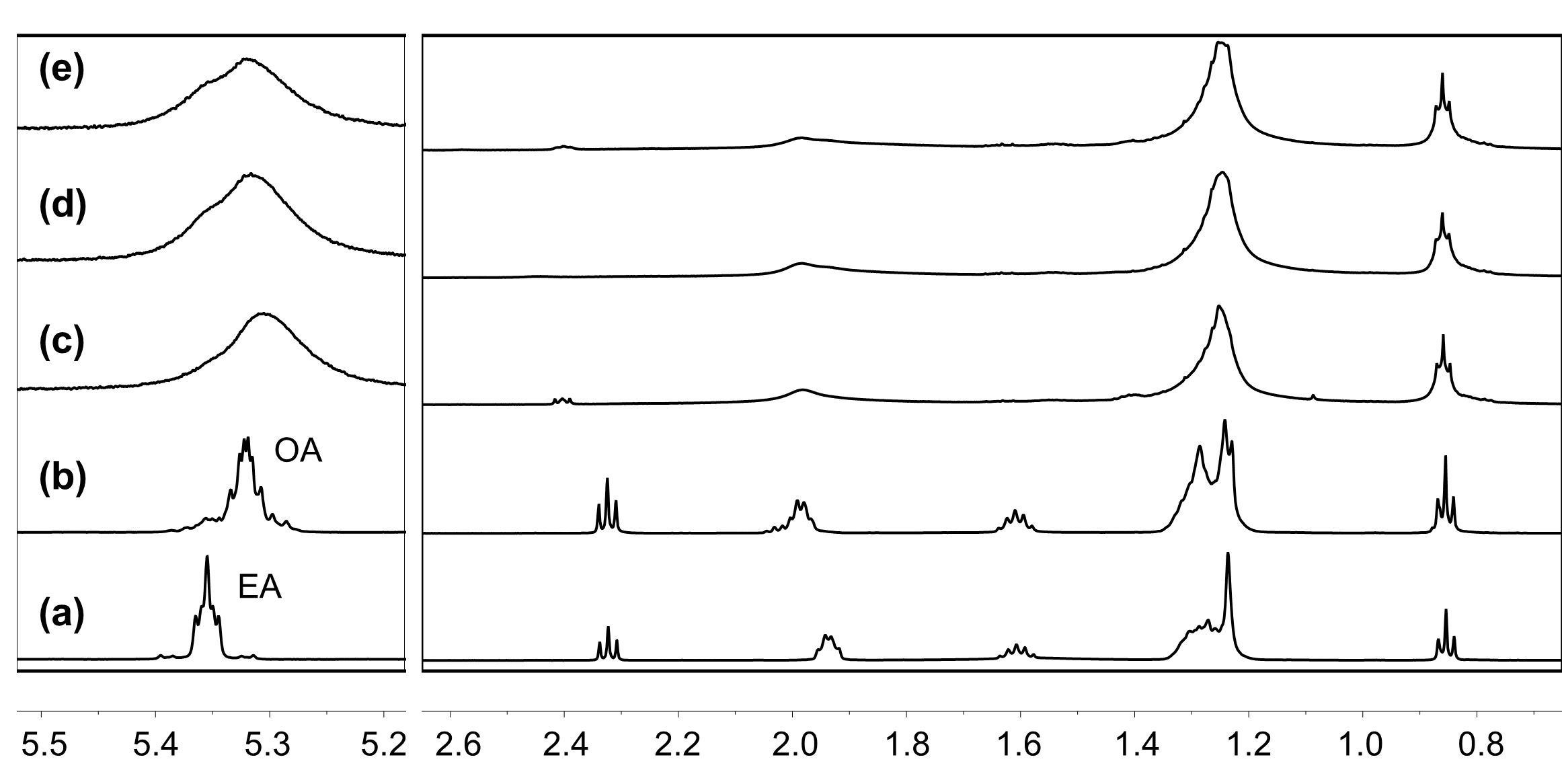


**Figure S8.** ^1^H NMR spectra of the as-prepared (without H_2_O_2_ treatment) samples in CDCl_3_. ^1^H NMR spectra of (a) elaidic acid (EA), (b) oleic acid (OA), (c) CdS-OA QD, (d) InP/ZnS-OA QD batch #1, and (e) InP/ZnS-OA QD batch #2 are compared. Note that the as-prepared InP/ZnS-OA QD samples exhibited the overlapped signal (~5.35 ppm) on the shoulder of the OA signal at ~5.3 ppm.

**
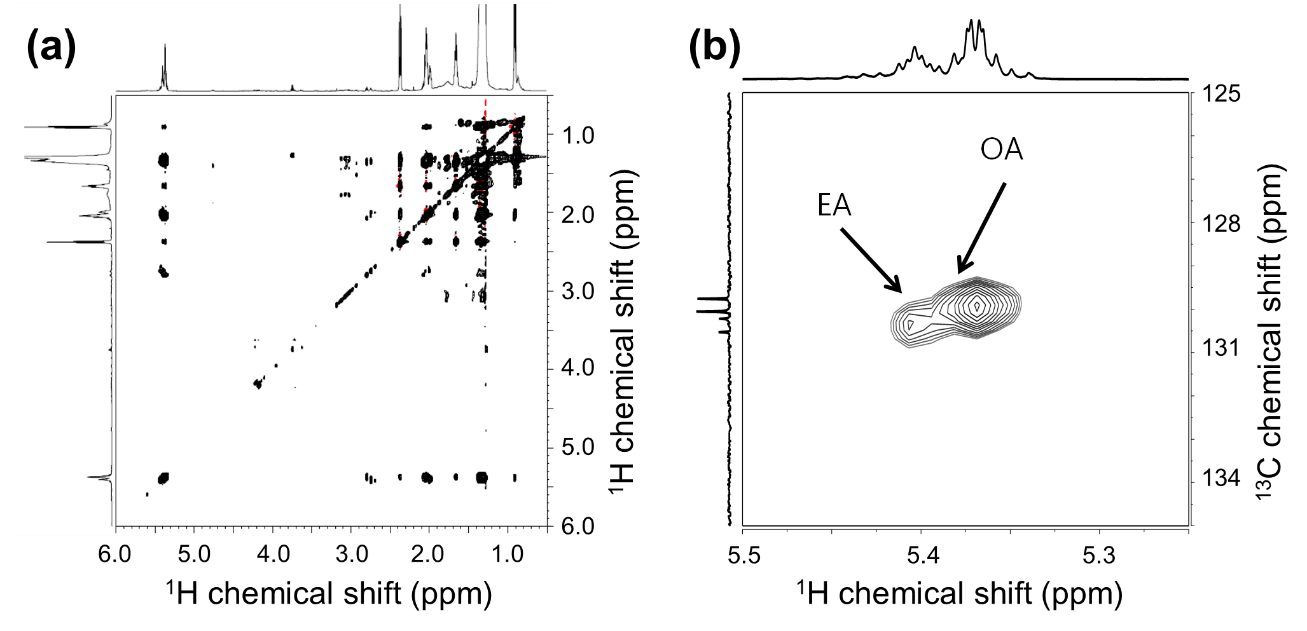
**

**Figure S9.** (a) ^1^H–^1^H TOCSY, (b) ^1^H–^13^C HSQC spectra of the CDCl_3_ fraction separated from the H_2_O_2_-treated InP/ZnS-OA. The extra peaks (marked as ‘EA’) near the signals of OA correspond to the *trans*-isomer of OA, elaidic acid (EA).

**Figure S10.** Extracted ion chromatograms of (a) OA and (b) the CDCl_3_ fraction of the H_2_O_2_-treated InP/ZnS-OA QD 1 sample, and (c) mass spectra of EA and OA. Although the mass spectra of EA and OA are nearly the same, they could be distinguished with a highly polar column that is able to detect the isomeric difference between the two molecules. Notably, the panel (a) indicates that the OA stock solution did not contain any other molecule except a trace amount of EA (~0.6 %).

**Figure S11.** The UV-Vis absorption and photoluminescence (PL) spectra (a) and the transmission electron microscopy image (b) of the as-prepared InP/ZnS-OA QD sample.

**Supplementary method**

**The surface coverage analysis procedure and results of InP/ZnS-OA QD samples**

1) Calibration with the standard solutions of OA (%w/w) in CDCl_3_ solution

| OA/CDCl_3_ | OA (g) | OA (mole) |
| --- | --- | --- |
| 0.74 | 0.0009 | 3.2×10^-6^ |
| 3.02 | 0.0027 | 9.6×10^-6^ |
| 5.02 | 0.0048 | 1.7×10^-6^ |
| 7.56 | 0.0072 | 2.6×10^-5^ |
| 9.38 | 0.0087 | 3.1×10^-5^ |

2) Calculation of the amount of OA per QD (mole/g) from the above calibration curve

| OA/CDCl_3_ | QD weight (g) | OA/QD (mole/g) |
| --- | --- | --- |
| 0.38 | 0.002 | 1.14×10^-6^ |
| 0.88 | 0.005 | 2.63×10^-6^ |
| 1.90 | 0.01 | 5.69×10^-6^ |
| 3.55 | 0.02 | 1.06×10^-5^ |
| 9.18 | 0.05 | 2.75×10^-5^ |

3) Calculation of the molecular weight of QDs from absorption signals, ICP-AES, and TGA.

From the relation between band gap and InP QD size, i.e., *E_g_*=1.401 + 3.493/*d*^1.172^ (*E_g_* (eV) is a band gap derived from the absorption peak, and *d* (nm) is the diameter of InP QD)^1^, the core size of InP was estimated to 2.5 nm. Using the atomic ratio measured with ICP-AES and the weight ratio of organic material from TGA, we could obtain the molecular weight and the size of InP/ZnS-OA QD.

■ Measured data

|  | Atomic Ratio | | | Weight Ratio |
| --- | --- | --- | --- | --- |
|  | P/In | S/In | Zn/In | Organics (wt.%) |
| InP/ZnS-OA QD | 0.85 | 4.20 | 6.20 | 32 |


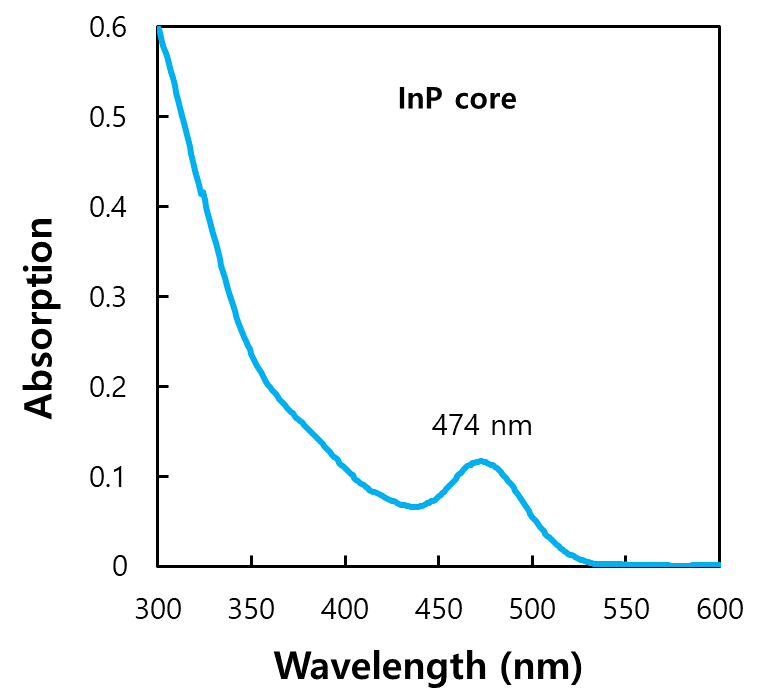


■ Result

|  | Size (nm) | | | Molecular Weight  (g/mole) |
| --- | --- | --- | --- | --- |
|  | InP (dia.) | ZnS (thick.) | QD Size (dia.) |  |
| InP/ZnS-OA QD | 2.5 | 0.8 | 4.1 | 130000 |

4) Estimation of the numbers of OA per QD and the surface coverage

Using the following relation OA/QD = 5.5×10^-4^ mole/g and a molecular weight of 130000, we could obtain the number of OA per QD and the surface coverage of OA on QD as 72 and 1.4/nm^2^, respectively.

**References**

1. Cho, E., Jang, H., Lee, J. & Jang, E. Modeling on the size dependent properties of InP quantum dots: a hybrid functional study. *Nanotechnology* **24**, 215201 (2013).
